# The impact of abusive supervision by coaches on athlete burnout in Chinese culture

**DOI:** 10.3389/fpsyg.2025.1643356

**Published:** 2025-09-18

**Authors:** Jingyan Li, Xingyi Li, JunJun Sun, Jiangyuan Li, Caixia Li, Yuantai Fu, Changliang Yan

**Affiliations:** ^1^Department of Sport and Leisure Studies, Namseoul University, Cheonan, Republic of Korea; ^2^Department of Sport and Health, Shinhan University, Uijeongbu, Republic of Korea; ^3^School of Foreign Language, Shandong Vocational and Technical University of International Studies, Rizhao, China; ^4^Department of Physical Education, Pukyong National University, Busan, Republic of Korea; ^5^Department of Physical Education, Taiyuan Institute of Technology, Taiyuan, China; ^6^Department of Physical Education, Northeastern University, Shenyang, China

**Keywords:** abusive supervision, athlete burnout, coach–athlete relationships, cognitive trust, affective trust

## Abstract

**Background:**

Empirical research on abusive supervision by coaches within the Chinese cultural context remains notably limited. Grounded in self-determination theory, the present study aims to explore the underlying mechanisms through which coach abusive supervision contributes to athlete burnout among Chinese athletes.

**Methods:**

This study employed a cross-sectional design and collected self-report questionnaire data from 301 athletes in China. A moderated mediation model was subsequently tested to examine the hypothesized relationships.

**Results:**

The findings indicate that the coach–athlete relationship mediates the association between abusive supervision and helping behavior. Moreover, a higher level of cognitive trust positively moderates the mediating effect of abusive supervision on the coach–athlete relationship, whereas the moderating role of affective trust is not statistically significant.

**Conclusion:**

The results indicate that as the level of coach abusive supervision increases, the quality of the coach–athlete relationship deteriorates, which in turn leads to heightened athlete burnout. Cognitive trust emerges as a significant moderating factor in this process. These findings suggest that fostering a supportive coach–athlete relationship—grounded in mutual respect and effective communication—remains a critical strategy for preventing athlete burnout.

## 1 Introduction

Among psychological and behavioral issues, burnout represents a significant concern for athletes and can exert serious negative effects on both athletic performance and athlete development (Maslach and Jackson, 1981). Research has shown that athletes experiencing severe burnout are significantly more likely to suffer from moderate to severe depression compared to those without such burnout (Kamimura et al., 2020). Notably, Chinese elite athletes typically begin specialized training at an earlier age and within closed training environments (Si et al., 2011). Compared to their Western counterparts, they are subjected to longer and more intensive training regimens and report higher levels of negative emotional experiences (Zhang et al., 2014).

In previous studies, leadership styles characterized by autonomy support, effective communication, and transformational behavior have been widely recognized for their positive impact on athlete burnout (Altahayneh, 2013; Bissett et al., 2020; Choi et al., 2020). Fransen et al. (2020) noted that high-quality leadership is positively related to the health of team members, suggesting that effective leadership can alleviate tendencies toward athlete burnout. However, not all coaches exhibit positive leadership behaviors toward athletes (Ely et al., 2010). There is still a lack of empirical research on the underlying mechanisms between negative coaching leadership and athlete burnout (Pacewicz et al., 2019).

Abusive supervision is defined as “supervisors’ persistent display of hostile verbal and non-verbal behaviors toward subordinates, excluding physical contact” (Tepper, 2000). Research shows that 22–25% of competitive athletes have experienced abusive supervision from their coaches in the sports domain (Stirling and Kerr, 2014). Influenced by Confucian values, Chinese culture emphasizes hierarchical superior–subordinate relationships, characterized by high power distance and traditional values (Hon et al., 2015). Within this context, team members tend to be highly sensitive to others’ expectations (Chiu, 1990), and subordinates in high-power-distance environments may be more susceptible to abusive behaviors from authority figures (Daniels and Greguras, 2014). Moreover, in collectivist cultures such as China, there is an emphasis on group harmony, interdependence, and prioritizing collective goals over individual needs (Zhao and Zhou, 2022). This cultural framework shapes the expectations and behaviors of both coaches and athletes, often leading to the normalization and acceptance of abusive coaching practices. Therefore, we argue that China represents an appropriate cultural setting for studying abusive supervision in sports. Although abusive supervision is a global phenomenon, prior research has predominantly focused on North American contexts, with limited empirical studies conducted in Chinese cultural settings (Pradhan et al., 2019; Zhang and Liao, 2015). This study seeks to address this important gap by examining the mechanisms through which abusive supervision by coaches contributes to athlete burnout in China.

This study adopts Self-Determination Theory (SDT) as a novel theoretical framework to explain the antecedents of athlete burnout. In previous research, scholars have applied Affective Events Theory (AET) to illustrate how abusive supervision negatively influences emotional states (Rani et al., 2021; Peng et al., 2019). Additionally, Social Exchange Theory (SET) has been used to explain the impact of abusive supervision on adverse employee behaviors through constructs such as interpersonal justice and psychological contract breach (Ghani et al., 2020; Khalid et al., 2018). The introduction of self-determination theory offers a fresh perspective by emphasizing the role of basic psychological needs in athlete development.

If all student-athletes were to break down under abusive coaching, it would be inevitable that such coaches would be compelled to alter their methods. However, why do some student-athletes appear to cope with abusive leadership more effectively than others, thereby allowing such behaviors to persist (Lopez et al., 2020)? A review of prior research on abusive leadership reveals that none of the antecedents examined to date have explored the role of trust in shaping the overall impact of abusive supervision. Therefore, we consider the moderating role of trust in the relationship between abusive supervision and athlete burnout.

## 2 Literature review and hypothesis development

### 2.1 Athlete burnout and abusive supervision by coaches

In 1974, Freudenberger introduced the concept of psychological fatigue to describe the responses of mental health professionals working in high-pressure environments (Fan et al., 2023). Subsequently, Maslach and Jackson (1981) proposed a theoretical model of burnout, characterizing it as the progressive depletion of psychological resources among individuals exposed to chronic occupational stress. This model consists of three core dimensions: emotional exhaustion, depersonalization, and a diminished sense of personal accomplishment. Although this framework has been effective in explaining how job-related stress influences psychological well-being in service-oriented industries, its applicability remains largely confined to “person–work” relationships, particularly in contexts involving provider–receiver interactions, and is more limited in non-service domains.

In the realm of sports, the pressures faced by athletes differ from those experienced by professionals in other occupational settings. Rather than focusing on interpersonal interactions, athletes primarily direct their attention toward personal performance goals (Raedeke and Smith, 2001). To address this distinction, Raedeke (1997) introduced the concept of athlete burnout, which captures athletes’ psychological responses to prolonged, high-intensity athletic demands. Athlete burnout is typically expressed through three key dimensions: (1) emotional and physical exhaustion, referring to the negative emotions and fatigue resulting from intense training and competition; (2) sport devaluation, wherein athletes lose interest and motivation toward their sport and may develop negative perceptions of it; and (3) a reduced sense of athletic accomplishment, in which athletes perceive their performance as falling short of expectations (Madigan et al., 2022). Recent studies have reported a growing prevalence of athlete burnout (Eklund and DeFreese, 2020). Its adverse consequences include diminished motivation, decreased engagement, impaired performance, strained interpersonal relationships, and an increased risk of depression. In severe cases, athletes may avoid training altogether or even permanently withdraw from sport participation (Larson et al., 2019). Therefore, further research into the mechanisms and influencing factors of athlete burnout is essential for safeguarding athletes’ mental health and informing the development of effective prevention strategies.

Abusive supervision refers to the sustained hostile verbal and non-verbal behaviors (excluding physical contact) that a superior (i.e., coach) directs toward subordinates (i.e., athletes), with the severity of these behaviors being subjectively perceived by the subordinate. Research shows that 22–25% of competitive athletes in the sports field have experienced abusive supervision from their coaches (Tepper, 2000). Some argue that the notion that “strict coaches produce top athletes” has led to the normalization and rationalization of this abusive behavior (Stirling and Kerr, 2014). However, other studies have shown that abusive supervision causes athletes to feel frustrated, stupid, and worthless (Stirling and Kerr, 2008). Abusive supervision by coaches makes athletes less tolerant and more emotional (Yukhymenko-Lescroart et al., 2015).

Research has demonstrated that abusive supervision by coaches—including verbal abuse, public humiliation, excessive criticism, and emotional neglect—exacerbates athletes’ psychological burnout (Cho et al., 2019; Mellano et al., 2022). In a longitudinal study of Swiss adolescent athletes, Gerber et al. (2024) found that when coaches exhibited emotionally abusive behaviors, such as yelling at athletes or engaging in derisive mockery, athletes showed a significant increase in burnout symptoms. Specifically, these athletes reported faster onset of emotional exhaustion, a loss of interest in training and competition, and even thoughts of withdrawing from sport altogether. From a motivational perspective, abusive supervision by coaches undermines athletes’ training motivation and enthusiasm (Zeng and Yin, 2025). According to Self-Determination Theory, when individuals’ basic psychological needs for autonomy, competence, and relatedness are persistently unmet, they are highly susceptible to burnout (Mageau and Vallerand, 2003). Coaches who engage in abusive supervision often adopt a highly controlling coaching style, depriving athletes of opportunities for autonomous decision-making and undermining their sense of competence through persistent denigration and negative feedback. For instance, when athletes perceive their coaches as excessively controlling and providing negative or ambiguous feedback, they tend to develop negative attitudes toward their coaches, experience heightened anxiety, and exhibit a marked decline in training motivation (Fransen et al., 2018).

China’s distinctive sports culture and management system have facilitated the emergence of abusive supervision by coaches and may profoundly influence athletes’ experiences of professional burnout. The core mechanism lies in the prevalence of a “paternalistic leadership” style among Chinese coaches, which embodies a duality of authoritarianism and benevolent care (Hou, 2024). Concurrently, under the traditional cultural ethos of “respecting teachers and valuing their authority” and the hierarchical governance of the “whole-nation system” (*juguo tizhi*), athletes are socialized from an early age to obey authority figures, often subordinating individual will to collective honor. When the authoritarian aspect of a coach’s leadership is excessively amplified and athletes lack the awareness or capacity to question and resist such authority, supervisory behaviors are highly susceptible to crossing boundaries and evolving into abusive practices. Such abusive behaviors not only provoke resistance from athletes and undermine team cohesion but may also constitute a critical contributing factor to professional burnout (Komaki and Tuakli-Wosornu, 2021). Nevertheless, empirical research focusing on the relationship between abusive supervision and athlete burnout within the context of Chinese sports remains scarce, highlighting a significant gap in the literature.

### 2.2 The mediating role of the coach–athlete relationship

Coaches and athletes are emotionally interconnected and mutually influential, making the coach–athlete relationship a unique interpersonal relationship that shapes both thoughts and behaviors (Jowett, 2009). This relationship extends beyond the competitive domain, evolving into a deep connection through psychological, emotional, and behavioral interactions. Smith defines the coach–athlete relationship as a multidimensional structure comprising closeness, commitment, and complementarity (the 3C model). Closeness reflects mutual trust, respect, and appreciation, as well as an inclination to like one another. Commitment denotes the readiness of coaches and athletes to consistently dedicate time and effort to sustaining their partnership, reflecting a shared expectation of relationship stability and continuity. Finally, complementarity indicates role complementarity and teamwork between coaches and athletes, especially during training (Smith, 1986).

Research has shown that abusive coaching behaviors damage the coach–athlete relationship: sustained verbal abuse and denigration rapidly erode athletes’ trust in their coaches, transforming what was once a cooperative instructional relationship into one marked by tension and antagonism (Ma et al., 2025; Marracho et al., 2023). Moreover, as the severity of abusive coaching escalates, athletes’ emotional distress tends to accumulate, and their negative perceptions of the coach become increasingly salient (Kim et al., 2020). When coaches’ hostile actions surpass a certain threshold, athletes may suffer from severe psychological exhaustion (ego depletion), perceiving their efforts as unrecognized and feeling helpless and emotionally drained. Such outcomes not only impair athletic performance but also trigger counterproductive behaviors such as retaliation or withdrawal from participation (Salehian and Mojtaba Sheikh Moghaddasi, 2021; Shannon et al., 2021).

A substantial body of research has confirmed that a positive coach–athlete relationship can alleviate athlete burnout, whereas a negative relationship may exacerbate the risk of burnout (Duhaylungsod et al., 2024; Fan et al., 2023; Gerber et al., 2024). When the coach–athlete relationship is of high quality, athletes are more likely to perceive care, understanding, and support from their coaches. Such social support is critical in buffering the stress associated with training and competition. As one of the most significant sources of social support for athletes, coaches play a vital role in helping athletes manage the demands of high-intensity training and rigorous competition. Emotional support and guidance from coaches can enable athletes to cope more effectively with stress and maintain both physical and psychological well-being (Duhaylungsod et al., 2024). Conversely, in the absence of adequate support and communication from coaches, athletes are more prone to feelings of isolation and helplessness. In highly demanding competitive environments, low levels of social support significantly increase the likelihood of athlete burnout. Moreover, research has indicated that when competitive pressure is high and coach support is lacking, athletes are more susceptible to emotional exhaustion and a sense of detachment from their sport (Ma et al., 2025). These findings underscore the protective role of social support provided by coaches in preventing burnout.

Self-Determination Theory (SDT) provides a critical lens for exploring the relationship between abusive coaching practices and athlete burnout. According to SDT, individuals’ healthy development depends on the satisfaction of three basic psychological needs: autonomy, competence, and relatedness, with coaches serving as a decisive external factor in this process (Mallett, 2005). In the sports context, a coach’s leadership style and the quality of the coach–athlete relationship directly influence the degree to which athletes’ basic psychological needs are either satisfied or thwarted. Abusive supervision, as a highly controlling and negative coaching style, fundamentally obstructs the fulfillment of athletes’ basic needs (Li et al., 2025). First, frequent verbal abuse and harsh criticism thwart athletes’ need for relatedness. Instead of feeling respected and accepted by their coach, athletes experience rejection and denigration, which erode the emotional bond between coach and athlete. Such thwarting of relatedness leaves athletes with a diminished sense of belonging and support, making them vulnerable to feelings of isolation and emotional exhaustion, thereby further undermining the quality of the coach–athlete relationship (Hagiwara et al., 2019). Second, when coaches intimidate athletes and suppress their autonomy through authoritative control, athletes are likely to feel a lack of agency, perceiving training as an obligation to endure rather than a process of self-directed engagement. This fosters helplessness and burnout. Moreover, persistent denigration and criticism can severely frustrate athletes’ need for competence. As athletes begin to doubt their own abilities and worth, they experience a loss of achievement and self-confidence, feelings of ineffectiveness that are widely recognized as a critical antecedent of burnout (Choi et al., 2020; Mellano et al., 2022). Therefore, based on self-determination theory, we predict that the coach–athlete relationship will mediate the relationship between abusive supervision by coaches and athlete burnout.

### 2.3 The moderating role of trust

Trust is primarily viewed as a psychological phenomenon. Myers et al. (2006) define trust as “the willingness of one party to be vulnerable to the actions of another party based on the expectation that the other will perform actions important to the trustor, regardless of the trustor’s ability to monitor or control the other”. Lewis and Weigert (1985) argue that all trust relationships encompass two dimensions: cognitive and emotional. They discuss cognitive trust, which involves knowledge, facts, and rational choice, and affective trust, which is based on shared principles, common values, emotions, and goodwill. Complementing the cognitive dimension of trust is the emotional dimension, which is grounded in emotional bonds and relational investment (Lewis and Weigert, 1985). When forming cognition, individuals often establish emotional ties with the object of trust, typically involving shared identity and values (Keating and Thrandardottir, 2017). The emotional dimension helps legitimize trust behavior based on a belief in the intrinsic virtues of the relationship (McAllister, 1995; Johnson and Grayson, 2005).

Additionally, in the cultural context of China, coaches often adopt a directive and instructive coaching style, which contrasts sharply with the more egalitarian coaching approaches in Western settings (Hodge and Lonsdale, 2011). This authoritative style creates a unique dynamic, fostering a strong trust in coaches among athletes. This trust, built through consistent and supportive coaching behavior, often leads athletes to make deep-seated commitments to training and performance (Lenartowicz, 2023). This also suggests that trust is a positive expectation and willingness by the trustor to accept a vulnerable position (Mayer et al., 1995). Through direct and repeated interactions between coach and athlete, if athletes believe the coach will fulfill commitments to the relationship, they are more likely to place greater trust in the coach (Chen and Wu, 2014).

In another study, Dirks (2000) surveyed athletes in NCAA college basketball teams in the United States and found that athletes’ trust in their coach affected team performance. Elsass (2001) verified the relationship between team performance and trust in the coach by dividing teams into high-performing and low-performing groups. The results showed that athletes in high-performing teams had a higher level of trust in their coach. Research indicates that when leaders and team members have a good relationship, they positively influence each other based on mutual trust and reciprocity, respecting and supporting those who benefit them (Zaker and Parnabas, 2018). Conversely, a poor leader–subordinate relationship can lead to a deterioration of the supervisor–subordinate relationship quality.

Social exchange theory posits that social behavior results from an exchange process (Bandura and Kavussanu, 2018). This relationship is often described as a form of reciprocity, where individuals seek to maximize benefits and minimize costs in interactions, reciprocating those who benefit them until the exchange is perceived as balanced. When coaches prioritize athletes’ best interests over their own, express care and concern, and create a trustworthy social environment, athletes’ perceived quality of the relationship with their coach tends to improve (Kao et al., 2017). Based on the above research and theories, we propose that affective trust and cognitive trust moderate the negative impact of abusive supervision by coaches on the coach–athlete relationship.

### 2.4 Current study

This study developed a moderated mediation model to examine the relationship between coaches’ abusive supervision and athlete burnout within the Chinese context. Four hypotheses were proposed: H1: There is a positive correlation between abusive supervision by coaches and athlete burnout. H2: The coach–athlete relationship mediates the relationship between abusive supervision by coaches and athlete burnout. H3: (a) Cognitive trust and (b) affective trust moderate the strength of the relationship between abusive supervision and athlete burnout. Additionally, we controlled for potential confounding variables, including athletes’ gender, age, competition level, and years of professional experience.

## 3 Method

### 3.1 Participants and procedure

This study employed a cross-sectional design and was approved by the local ethics committee (Approval No. SVTUIS202040528). Participants were recruited in two phases. In the first phase, data were collected from 12 top-tier universities in China using a convenience sampling method based on established research collaborations. In the second phase, a complete list of registered student-athletes was obtained from the sports departments, from which 334 athletes were randomly selected and invited to participate in the study. Our researchers then met with the athletes in person, with the assistance of the administrators, to explain the procedures for completing the questionnaire and the purpose of the study. We assured them of the confidentiality of the questionnaire. To reduce potential social desirability bias, the athletes submitted their completed questionnaires directly to the researchers rather than to department heads. These institutions represent the highest level of collegiate athletic competition in the country, largely due to China’s distinctive “high-performance athlete recruitment” policy. Under this national policy, elite universities are authorized to significantly lower their academic admission standards to enroll athletes who have already achieved high competitive rankings. These athletes’ primary responsibility during their university years is to represent their institutions in premier national competitions. Athletes in this context often face immense performance pressure, rigorous training schedules, and high-stakes relationships with their coaches. This unique environment is highly relevant to the core constructs examined in this study. Therefore, we consider this population to be an ideal sample for testing our theoretical model, as the constructs under investigation are likely to be more pronounced and observable in such a high-pressure context.

The questionnaire was completed using a paper-and-pencil method and conducted in three rounds, with a three-week interval between each round. Podsakoff et al. (2003) emphasize that time intervals are crucial in data collection. The intervals should be appropriate; too long an interval may weaken the relationships between variables, while too short an interval may exaggerate them. Therefore, a three-week interval was considered ideal (Dormann and Griffin, 2015). Respondent confidentiality was ensured. Each respondent was assigned a unique identifier to match responses across the three rounds without involving personal identifying information. In the initial phase (T1), survey invitations were sent to 334 athletes, of whom 327 completed the questionnaire, yielding an initial response rate of 97.9%. Data collected at T1 included demographic information and the independent variable, abusive supervision. In the subsequent phase (T2), follow-up surveys were distributed to T1 participants to collect data on the moderating variable (cognitive trust) and the mediating variable (coach–athlete relationship). A total of 320 participants responded at this stage. In the final phase (T3), surveys assessing the dependent variable—professional burnout—were administered to athletes who had responded at T2, resulting in 306 responses. The overall retention rate from T1 to T3 was 93.6%, corresponding to a natural attrition rate of 6.4%. After excluding five outlier questionnaires, 301 valid responses were retained for analysis. Participants’ demographic characteristics are presented in Table 1.

**Table 1:** Demographic information of the respondents.

Variables	Categories	Frequency	(%)
Gender	Male	157	52.2
	Female	144	47.8
Age	<20	43	14.3
	20–23	205	68.1
	>23	53	17.6
	<7	85	28.2
Career	8–10	128	42.6
	>10	88	29.2
National team experience	YES	12	4.0
	NO	288	96.0
Family size	3	57	19.0
	4	137	45.6
	5	70	23.2
	>5	37	12.3

To determine the necessary sample size, G-power 3.1.9.7 was utilized, assuming an alpha level of 0.05, medium effect size (*f*^2^) of 0.15, statistical power (1−*β*) of 0.80, and six predictors (including one independent variable, mediator, two moderators, and two interaction terms), indicating a minimum requirement of 96 participants. Thus, the final sample of 301 was considered sufficient for model testing.

### 3.2 Measures

The constructs in this study are based on previous research. All items, except for those measuring abusive supervision, were assessed on a 7-point Likert scale (7 = strongly agree, 1 = strongly disagree), while abusive supervision and athlete burnout items were measured using a 5-point Likert scale (5 = strongly agree, 1 = strongly disagree).

#### 3.2.1 Abusive supervision

Subordinates’ perceptions of abusive supervision were measured using a five-item abusive supervision scale (Tepper, 2000). Items such as “My supervisor ridicules me” and “My supervisor says my ideas and feelings are stupid” were assessed on a Likert scale.

#### 3.2.2 Coach–athlete relationship

The 11-item French version of the Coach–Athlete Relationship Questionnaire (CART-Q) was used to assess athletes’ perceptions of the quality of the coach–athlete relationship (Jowett and Ntoumanis, 2004). The CART-Q measures the quality of this relationship across three interpersonal dimensions (the 3C model): closeness (4 items; measures the emotional bond between athlete and coach, e.g., “I like my coach”), commitment (3 items; measures commitment to working with the coach long-term, e.g., “I am committed to my coach”), and complementarity (4 items; measures the level of cooperation between athlete and coach, e.g., “When I receive guidance from my coach, I am ready to do my best”).

#### 3.2.3 Burnout

The Athlete Burnout Questionnaire, designed by Raedeke and Smith (2001), includes three dimensions: emotional/physical exhaustion (e.g., “I feel overly exhausted after participating in my sport”), reduced sense of athletic achievement (e.g., “It seems that no matter what I do, I cannot do my best”), and sport devaluation (e.g., “The energy I spend on training and competition would be better spent doing other things”).

#### 3.2.4 Trust

Cognitive trust was measured using the supervisor trust scale by Yang and Mossholder (2010), with six items (e.g., “I can rely on my supervisor to do what is best for my work”). Affective trust was assessed using items from the supervisor trust scale by Yang and Mossholder (2010), with five items (e.g., “I believe my supervisor genuinely cares about my personal needs at work”).

### 3.3 Data analysis

Data analysis was conducted using various statistical tools in SPSS and MPLUS. Composite reliability (CR), factor loadings, Cronbach’s alpha, and average variance extracted (AVE) were assessed to evaluate the reliability and validity of the research model. Confirmatory factor analysis (CFA) was also performed to determine model fit indices, such as λ ^2^ (CMIN/df), root mean square error of approximation (RMSEA), incremental fit index (IFI), comparative fit index (CFI), and Tucker-Lewis index (TLI).

**Figure 1:**
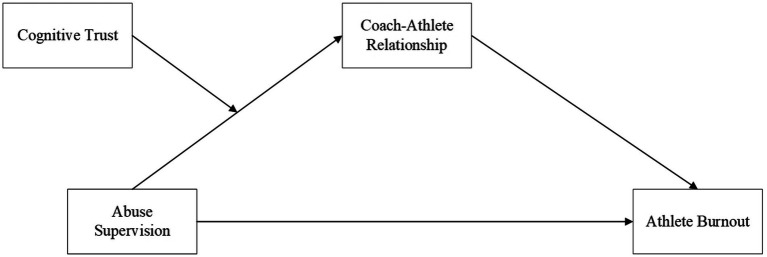
Hypothesized model 1.

**Figure 2:**
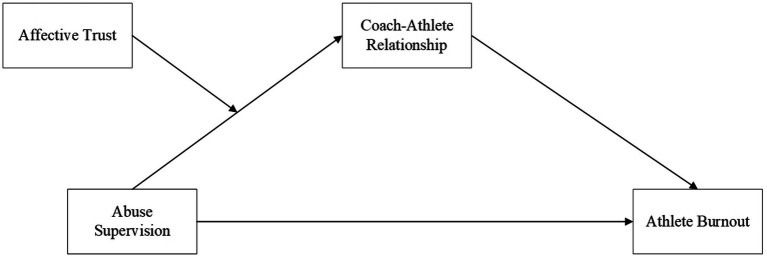
Hypothesized model 2.

Hypotheses were tested in two interrelated steps. First, hierarchical regression analysis was conducted using Baron and Kenny’s (1986) simple mediation model (Hypotheses 1 and 2). Second, we employed the PROCESS macro (Bond et al., 2011) to test moderation and mediation effects. Bootstrap analysis via path analysis procedures was conducted to examine the indirect effect hypotheses and their significance (Edwards and Lambert, 2007). Bootstrap is a reliable and advanced method for estimating indirect effects in social sciences (Montoya and Hayes, 2017). Through these procedures, we demonstrated the mediating (indirect) effect of the athlete–coach relationship on the association between abusive supervision and burnout. In addition, we conducted a Johnson-Neyman technique analysis to examine the moderating effect and plotted simple slope graphs at one standard deviation above and below the moderator. Prior to the main analyses, we screened the data for miscoding, outliers, missing values, and multicollinearity, and no such issues were identified in our dataset (see Figures 1, 2).

## 4 Results

Before testing the hypothesized relationships, we first conducted confirmatory factor analyses (CFAs) on the proposed model using the MPLUS software package to ensure construct distinctiveness among the study variables. To ensure sufficient degrees of freedom, the five primary research variables were segmented within the model. The resulting five-factor model showed an excellent fit with the data [ χ ^2^(1,258) = 1603.964, CFI = 0.936, TLI = 0.933, RMSEA = 0.030].

In line with recommendations from previous studies, we assessed reliability and validity using Cronbach’s *α*, AVE, CR, and factor loadings. As presented in Table 2, Cronbach’s *α*, CR, and AVE values meet the thresholds of 0.70, 0.60, and 0.50, respectively. Convergent validity for each construct was evaluated through factor loading values, with Table 2 showing that all item loadings exceed the 0.70 thresholds, indicating strong convergent validity. Discriminant validity was further examined based on Fornell and Larcker’s (1981) criterion, which recommends that inter-construct correlations should be lower than the square root of the AVE for each construct. Table 2 confirms that all construct correlations fall below the square root of their respective AVE values, affirming the model’s discriminant validity.

**Table 2:** Reliability and validity measurement.

Latent variable	Observed variable	SC	Cronbach’s *α*	CR	AVE
Abusive supervision (AS)	AS1	0.650			
	AS2	0.661			
	AS3	0.694			
	AS4	0.734			
	AS5	0.693			
	AS6	0.675			
	AS7	0.743			
	AS8	0.723	0.936	0.936	0.500
	AS9	0.741			
	AS10	0.712			
	AS11	0.703			
	AS12	0.695			
	AS13	0.745			
	AS14	0.716			
	AS15	0.691			
Coach–athlete relationship	Closeness	0.792			
	Commitment	0.862	0.834	0.844	0.648
	Complementarity	0.671			
Athlete burnout	Reduced sense of accomplishment	0.834			
	Emotional and physical exhaustion	0.686	0.785	0.776	0.539
	Sport devaluation	0.817			
Cognitive trust (CT)	CT1	0.747			
	CT2	0.764			
	CT3	0.704	0.875	0.876	0.542
	CT4	0.682			
	CT5	0.738			
	CT6	0.778			
Affective trust (AT)	AT1	0.676			
	AT2	0.721			
	AT3	0.774	0.825	0.826	0.488
	AT4	0.669			
	AT5	0.647			

Table 3 lists the correlations between variables, which are significant and consistent with expectations. Table 4 presents the regression analysis of the mediating effect. According to Baron and Kenny’s (1986) mediation criteria (1) the independent variable must have a significant relationship with the dependent variable; (2) the independent variable must significantly affect the mediator; (3) the mediator must be significantly associated with the dependent variable when controlling for the independent variable. Hypothesis 1 meets the first criterion (*β* = −0.522, *p* < 0.01), and the relationship between abusive supervision and the coach–athlete relationship meets the second criterion (*β* = −0.154, *p* < 0.001). For the third criterion, regression analysis indicates a significant relationship between the coach–athlete relationship and burnout (*β* = −0.31, *p* < 0.01), which reduces the impact of abusive supervision on burnout (*β* = 0.504, *p* < 0.05).

**Table 3:** Means, standard deviations, reliabilities, and correlations among variables.

	Variable	M	SD	AS	CAR	AB	AT	CT
1	AS	27.179	8.400	1				
2	CAR	37.322	7.735	−0.152^**^	1			
3	AB	25.152	6.863	0.536^**^	−0.203^**^	1		
4	AT	20.425	6.513	0.064	0.47	0.147^*^	1	
5	CT	37.382	5.849	0.164^**^	0.122^*^	0.030	0.063	1

AS, abusive supervision; CAR, coach–athlete relationship; AB, athlete burnout; CT, cognitive Trust; AT, affective trust.

*N* = 301, ^*^p < 0.05, ^**^p < 0.01, ^***^p < 0.001 (two-tailed).

**Table 4:** Hierarchical regression results for simple mediation.

	Coach–athlete relationship		Athlete burnout		
	Step1 (β)	Step2 (β)	Step1 (β)	Step2 (β)	Step3 (β)
Step1. Control variables^a^					
Age	0.057	0.051	−0.042	−0.019	−0.013
Gender	0.009	0.011	0.000	−0.008	−0.006
Career year	−0.034	−0.021	0.076	0.031	0.028
National team experience	0.028	0.01	−0.189	−0.128^**^	−0.127^**^
Family size	−0.045	−0.06	0.003	0.055	0.047
Step 2. Main effect					
Abusive Supervision		−0.154^**^		0.522^***^	0.504^***^
Step 3. Main effect					
Coach–athlete relationship					−0.310^***^
Overall *F*	0.442	1.523	2.721	21.694	19.775
*R* ^2^	0.007	0.030	0.044	0.307	0.321
Δ *F*	0.442	6.883	2.721	111.467	6.033
Δ *R* ^2^	0.007	0.023	0.044	0.263	0.014

Bootstrap results for indirect effect				
	Effect	SE	LL, 95% CI	UL, 95% CI
Indirect effect	0.018	0.012	0.002	0.048

*N* = 301, Bootstrap sample size = 10,000, LL, lower limit; CI, confidence interval; UL, upper limit.

^*^p < 0.05, ^**^p < 0.01, ^***^p < 0.001 (two-tailed).

Entries are standardized regression coefficients.

a Variables are standardized variables.

A 95% bias-corrected confidence interval (CI) was generated by bootstrapping 10,000 samples to test the significance of the indirect effect. The Bootstrap analysis showed a CI of 0.002 to 0.048, thus supporting Hypothesis 2.

**Figure 3:**
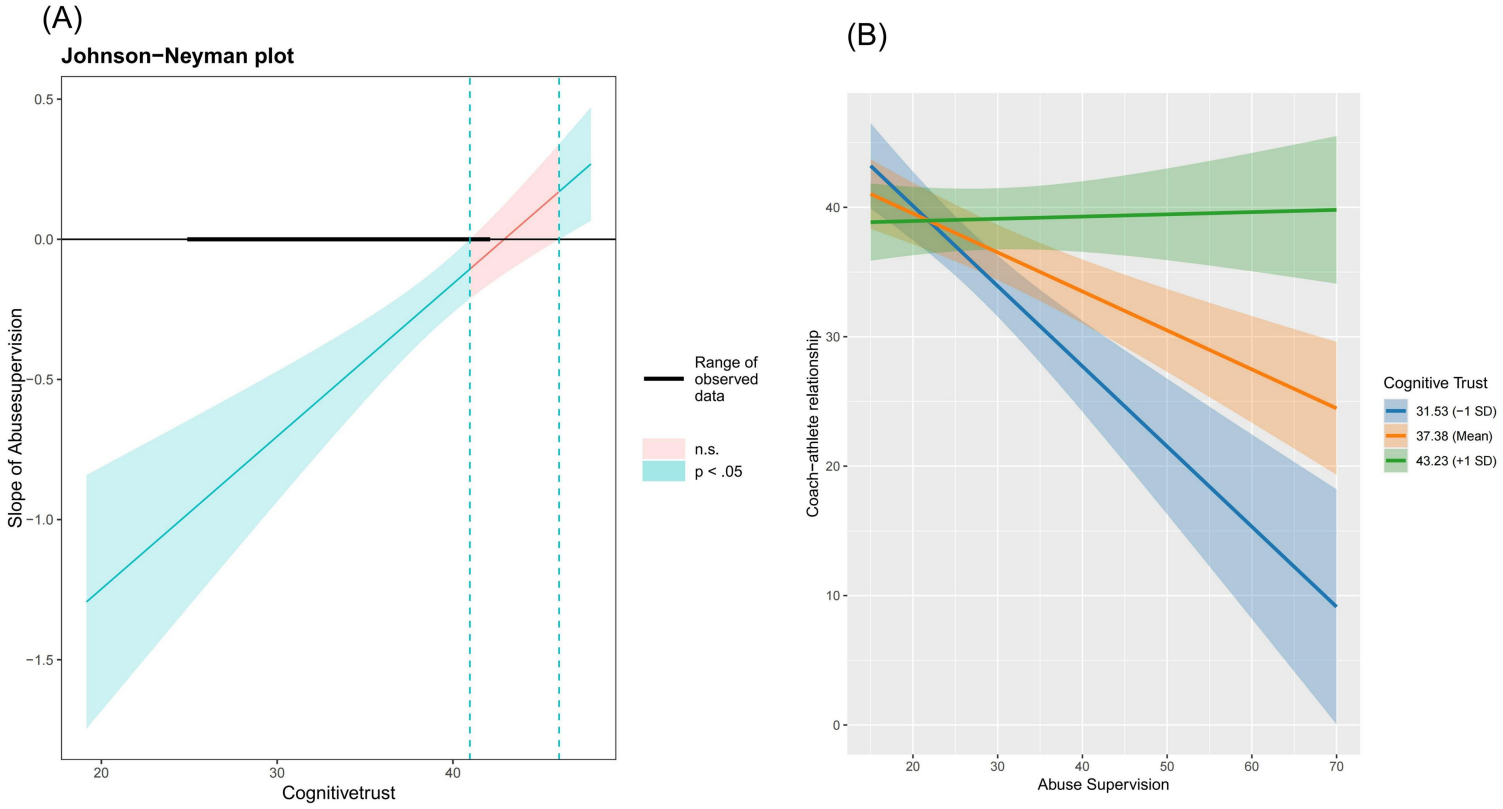
Moderating effects test. **(A)** The Johnson–Neyman plot. **(B)** Simple slope analysis of the interaction between cognitive trust and abusive supervision on the coach–athlete relationship.

Table 5 presents the regression analysis of the moderating effect. Hypothesis 3 proposed that both high affective trust (3a) and high cognitive trust (3b) would weaken the indirect effect of the coach–athlete relationship on the link between abusive supervision and burnout. However, the interaction between affective trust and abusive supervision did not significantly moderate the coach–athlete relationship (*β* = −0.421, *p* = n.s), thus rejecting Hypothesis 3a. Conversely, the interaction between abusive supervision and cognitive trust was significant (*β* = 0.312, *p* < 0.05). To further clarify this interaction, simple slopes were plotted at one standard deviation above and below the mean of cognitive trust (Figure 3B). As anticipated, Athletes with low levels of cognitive trust had a larger slope than those with high levels of cognitive trust.

**Table 5:** Hierarchical regression results for moderated mediation.

	Coach–athlete relationship			
	Step1 (β)	Step2 (β)	Step3 (β)	Step4 (β)
Step1. Control variables^a^				
Age	0.057	0.051	0.042	0.018
Gender	0.009	0.011	0.018	0.021
Career year	−0.034	−0.021	−0.018	−0.031
National team experience	0.028	0.01	0	−0.025
Family size	−0.045	−0.06	−0.106	−0.075
Step 2. Main effect				
Abusive supervision		−0.154^**^	−0.193^***^	−0.335^***^
Step 3. Main effect				
Cognitive trust			0.171^**^	0.217^***^
Affective trust			0.06	0.069
Step 4. Moderating effect				
Abusive supervision ∗ CT^b^				0.312^***^
Abusive supervision ∗ AT^c^				−0.421
Overall *F*	0.442	1.523	2.372	4.593
*R* ^2^	0.007	0.030	0.061	0.137
Δ *F*	0.442	6.883	4.802	12.718
Δ *R* ^2^	0.007	0.023	0.031	0.076

*N* = 301, bootstrap sample size = 10,000. LL, lower limit; CI, confidence interval; UL, upper limit.

^*^p < 0.05, ^**^p < 0.01, ^***^p < 0.001.

Entries are standardized regression coefficients.

a Variables are standardized variables.

b CT, cognitive trust.

c AT, affective trust.

We employed the Johnson-Neyman technique to examine the conditional indirect effect of abusive supervision on burnout (Figure 3A). The results indicated that the slope of abusive supervision was significant when cognitive trust fell outside the range of [40.96, 46.04], suggesting a significant moderating effect of cognitive trust on the relationship between coach abusive supervision and athlete burnout. Therefore, Hypothesis 3a was supported.

## 5 Discussion

Athlete burnout has profound effects on athletes’ mental health, athletic performance, and overall well-being. As the primary influence in athletes’ social environments, coaches play a crucial role in predicting levels of athlete burnout. Previous studies have widely recognized that positive leadership styles and behaviors, characterized by autonomy support, effective communication, and transformational behavior, positively impact athlete burnout (Altahayneh, 2013; Bissett et al., 2020; Choi et al., 2020). However, the underlying mechanisms between coaches’ negative leadership and athlete burnout remain understudied. Based on self-determination theory, this study examined the impact of abusive supervision by coaches on athlete burnout within a Chinese context, focusing on the mediating role of the coach–athlete relationship and the moderating role of trust.

The present study found a significant association between coaches’ abusive supervision and athletes’ burnout. Specifically, the more frequently and severely athletes perceived abusive coaching behaviors, the higher their overall levels of professional burnout. This finding aligns with previous research (Duhaylungsod et al., 2024; Fan et al., 2023; Gerber et al., 2024), which suggests that abusive supervision, as a potent interpersonal stressor, persistently depletes athletes’ psychological resources such as self-esteem and emotional stability. When athletes remain in such a state of resource depletion over time, they are more likely to experience increased fatigue and feelings of detachment—hallmark symptoms of burnout. This result contributes additional evidence to the harmful psychological effects of abusive coaching practices within the Chinese cultural context.

The findings indicate that the coach–athlete relationship mediates the association between coaches’ abusive supervision and athletes’ burnout. Specifically, as abusive supervision intensifies, the quality of the coach–athlete relationship deteriorates, which in turn exacerbates athletes’ professional burnout. This result aligns with previous research (Duhaylungsod et al., 2024; Fan et al., 2023; Gerber et al., 2024), showing that when coach–athlete relationships are harmonious, characterized by mutual trust and effective communication, athletes tend to feel supported and a sense of belonging, which is associated with significantly lower levels of burnout. Conversely, when relationships are strained and emotionally distant, athletes are more vulnerable to burnout. Abusive supervision may erode trust and closeness within the coach–athlete relationship, depriving athletes of a vital source of psychological support and thereby contributing to burnout. Moreover, this mediating pathway is echoed in the broader field of organizational management, where abusive leadership has been shown to undermine subordinates’ trust in their leaders, reduce relationship quality, and trigger a range of negative outcomes, including job burnout (Liao et al., 2021; Nevicka et al., 2018). This mechanism may be particularly salient in the Chinese cultural context, where relationship-oriented values (*guanxi benwei*) are deeply embedded. Compared to athletes in individualistic cultures, Chinese athletes rely more heavily on harmonious relationships with their coaches for psychological safety and motivation (Zhang et al., 2014). As such, the disruption of these relationships may have even more pronounced adverse effects.

By introducing an organizational factor—trust—as a means to mitigate the negative effects of leader abuse of power, this study addresses a gap in the literature. Our research examined cognitive and affective trust as two moderating factors that, through the coach–athlete relationship, alleviate the adverse impact of abusive supervision on athlete burnout. A noteworthy finding is that athletes’ cognitive trust moderates the relationship between coaches’ abusive supervision and the coach–athlete relationship. Specifically, when athletes hold a high level of cognitive trust in their coaches, the damaging effect of abusive behaviors on relationship quality is “buffered,” which helps maintain the stability of the coach–athlete relationship to some extent. This is consistent with prior studies (Jiahao and Jing, 2024; Lenartowicz, 2023), which suggest that under high cognitive trust, athletes firmly believe that their coaches’ intentions are benevolent and aimed at their best interests, making them more willing to tolerate a strict and demanding coaching style. This mechanism resonates with the traditional Chinese notion of “strict teachers produce outstanding students” (*yan shi chu gao tu*). When athletes are convinced that their coaches’ harshness is driven by goodwill and sophisticated training intentions, they are more likely to interpret criticism as an expression of care rather than as a hostile attack (Jiahao and Jing, 2024; Kim and Choi, 2024). However, affective trust did not have a statistically significant moderating effect on the relationship between abusive supervision and burnout. Several factors may explain the differing moderating roles of affective and cognitive trust. First, social exchange theory suggests that social behavior results from an exchange process (Bandura and Kavussanu, 2018). This relationship is often described as a form of reciprocity, where individuals seek to maximize benefits and minimize costs in interactions, reciprocating those who benefit them until a balanced exchange is perceived. Cognitive trust is based on a rational assessment of the coach’s competence and reliability (Srivastava et al., 2006). Therefore, when athletes perceive that their coach’s knowledge can help them achieve better performance, they may use self-regulation and cues to reduce stress and maintain a positive relationship with the coach. Second, abusive management can cause severe emotional harm to athletes, leading to a depletion of emotional resources and, ultimately, emotional exhaustion (Cropanzano et al., 2003). Emotionally exhausted individuals reduce their efforts in contextual performance dimensions, such as interpersonal facilitation (Bartol et al., 2009). It is worth noting that in this study, Social Exchange Theory offers an important complementary perspective. The exchanges between coaches and athletes do not alter the basic needs described by SDT; rather, Social Exchange Theory serves as a cognitive and emotional filter that shapes athletes’ perceptions of the exchanges themselves. This perspective helps explain how athletes’ perceptions of long-term relational balance and fairness influence their interpretations of objectively negative behaviors. Essentially, these two theories operate in a complementary manner, providing a more comprehensive understanding of the complex interplay between coach abusive supervision, athlete burnout, and the relational context.

Our findings possess numerous practical implications. First, coaches’ behavior may be a significant factor influencing athlete burnout. Although sports organizations invest in preventing athlete burnout, their efforts may be ineffective if leaders abuse their power. Considering the detrimental effects of abusive supervision, sports organizations should implement targeted policies and allocate resources to curb such behavior among coaches. Our findings suggest that organizations should prioritize enhancing coaches’ professional competencies to build athletes’ cognitive trust, thereby mitigating the adverse impacts of abusive supervision. Providing athletes with more social support and resources can help lessen the negative effects of abusive behavior by coaches. Furthermore, efforts should be made to reduce the stress level in athletes’ training and competition environments. Improving coach effectiveness, including interpersonal skills (such as effective communication, active listening, care, and understanding), personal knowledge (such as motivation and self-confidence), and relational constructs like trust, connection, and respect, can enhance athletes’ perception of the coach–athlete relationship, as this relationship positively impacts athlete burnout. Sports organizations should create a harmonious interpersonal or social environment for athletes. In such an environment, strong personal and emotional bonds of care and support, both in the short-term (here and now) and long-term (future), contribute to fostering a positive coach–athlete relationship.

## 6 Limitations and future research

Our study has several limitations that indicate directions for future research. Firstly, as the data were collected exclusively from university athletes, the findings may not be generalizable to high school or middle school athletes. Future research could expand the sampling scope to include these groups, thereby establishing a more comprehensive dataset. Moreover, as indicated by our data, the vast majority of participants (96.0%) lacked national team experience. This suggests that our findings are more representative of high-level sub-elite university athletes rather than top-tier national-level athletes. Future studies should aim to include more diverse samples across different competitive levels. Secondly, in the data collection process, we did not distinguish between athletes participating in individual sports and those in team sports. The dynamics of the coach–athlete relationship and manifestations of abusive supervision may differ between these two contexts. Therefore, the findings of this study reflect only the general trends observed in a mixed sample of high-level athletes. Future research is urgently needed to investigate and compare the mechanisms linking abusive supervision and burnout in individual versus team sport environments, in order to develop a more nuanced understanding of these relationships. Finally, future research could examine the influence of abusive coaching on other forms of psychological stress in athletes, such as anxiety and emotional exhaustion.

## 7 Conclusion

This study, situated within the Chinese cultural context, explored the underlying mechanisms linking coaches’ abusive supervision to athletes’ professional burnout. The findings revealed that as the degree of abusive supervision by coaches intensifies, the quality of the coach–athlete relationship deteriorates, thereby contributing to athletes’ professional burnout. Cognitive trust emerged as an important moderating factor, buffering the negative impact of abusive supervision on the coach–athlete relationship, whereas affective trust did not exhibit a significant moderating effect. These findings carry important practical implications for sports management departments, coaches, and athletes. Sports organizations should implement clear policies to identify and curb abusive coaching practices. Furthermore, efforts should be made to enhance coaches’ professional competencies and foster athletes’ cognitive trust, thereby mitigating some of the adverse effects of a strict coaching style. Ultimately, cultivating a supportive coach–athlete relationship grounded in respect and effective communication remains a key strategy for preventing athlete burnout.

## Data Availability

The original contributions presented in the study are included in the article/supplementary material, further inquiries can be directed to the corresponding authors.
